# Outlines of Practical Surgery

**Published:** 1899-03

**Authors:** 


**Outlines of Practical Surgery.** By WALTER G. SPENCER, M.B., M.S. Pp. x., 694. London: Baillière, Tindall and Cox. 1898.

The practical surgeon of the present day will be much relieved to find that the author has produced a work of such excellence, without going too deeply into the details of theoretical pathology and bacteriology.

The diagrammatic nature of the illustrations with which the various chapters abound are most clear, and by examining these "black and white figures" the student of practical surgery can readily comprehend numerous details in the operative art, which in some books are not too clearly explained in the text.

We are sorry to see in the pages devoted to bandaging that no mention is made of the good old method of "reversing" in spiral bandaging, and we do not quite agree with the text as to where the spiral bandage may be used. It is certainly doubtful whether (if we except the joints) the commonest method of bandaging is the figure-of-eight. We do not like the description of the suturing of the two ends of the divided intestine round the male and female portions of Murphy's button; the approved method might, in a future edition of the book, be clearly demonstrated by a diagram.

On reading the volume through we find little of importance to detract from its value, and we recommend it as a most useful addition to the library of those wanting a practical book on surgery.

